# Immune Reconstitution-Based Score for Risk Stratification of Chronic Graft-Versus-Host Disease Patients

**DOI:** 10.3389/fonc.2021.705568

**Published:** 2021-07-22

**Authors:** Fabio Serpenti, Francesca Lorentino, Sarah Marktel, Raffaella Milani, Carlo Messina, Raffaella Greco, Stefania Girlanda, Daniela Clerici, Fabio Giglio, Carmine Liberatore, Francesca Farina, Sara Mastaglio, Simona Piemontese, Elena Guggiari, Francesca Lunghi, Magda Marcatti, Matteo G. Carrabba, Massimo Bernardi, Chiara Bonini, Andrea Assanelli, Consuelo Corti, Jacopo Peccatori, Fabio Ciceri, Maria Teresa Lupo-Stanghellini

**Affiliations:** ^1^Hematology and Bone Marrow Transplantation Unit, IRCCS San Raffaele Scientific Institute, Milan, Italy; ^2^PhD Program in Public Health, School of Medicine and Surgery, University of Milano Bicocca, Milan, Italy; ^3^Immunohematology and Transfusion Medicine Unit, IRCCS San Raffaele Scientific Institute, Milan, Italy; ^4^University Vita-Salute, Milan, Italy; ^5^Experimental Hematology Unit, IRCCS San Raffaele Scientific Institute, Milan, Italy

**Keywords:** chronic GvHD, immune reconstitution, biomarker, prognostic score, overall survival

## Abstract

**Introduction:**

Allogeneic stem cell transplantation survivors are at a relevant risk of developing chronic GvHD (cGvHD), which importantly affects quality of life and increases morbidity and mortality. Early identification of patients at risk of cGvHD-related morbidity could represent a relevant tool to tailor preventive strategies. The aim of this study was to evaluate the prognostic power of immune reconstitution (IR) at cGvHD onset through an IR-based score.

**Methods:**

We analyzed data from 411 adult patients consecutively transplanted between January 2011 and December 2016 at our Institution: 151 patients developed cGvHD (median follow-up 4 years). A first set of 111 consecutive patients with cGvHD entered the test cohort while an additional consecutive 40 patients represented the validation cohort. A Cox multivariate model for OS (overall survival) in patients with cGvHD of any severity allowed the identification of six variables independently predicting OS and TRM (transplant-related mortality). A formula for a prognostic risk index using the β coefficients derived from the model was designed. Each patient was assigned a score defining three groups of risk (low, intermediate, and high).

**Results:**

Our multivariate model defined the variables independently predicting OS at cGvHD onset: CD4+ >233 cells/mm^3^, NK <115 cells/mm^3^, IgA <0.43g/L, IgM <0.45g/L, Karnofsky PS <80%, platelets <100x10^3^/mm^3^. Low-risk patients were defined as having a score ≤3.09, intermediate-risk patients >3.09 and ≤6.9, and high-risk patients >6.9. By ROC analysis, we identified a cut-off of 6.310 for both TRM and overall mortality.

In the training cohort, the 6-year OS and TRM from cGvHD occurrence were 85% (95% CI, 70-92) and 13% (95% CI, 5-25) for low-risk, 64% (95% CI, 44-89) and 30% (95% CI, 15-47) for intermediate-risk, 26% (95% CI, 10-47), and 42% (95% CI, 19-63) for high-risk patients (OS p<0.0001; TRM p = 0.015).

The validation cohort confirmed the model with a 6-year OS and TRM of 83% (95% CI, 48-96) and 8% (95% CI, 1-32) for low-risk, 78% (95% CI, 37-94) and 11% (95% CI, 1-41) for intermediate-risk, 37% (95% CI, 17-58), and 63% (95% CI, 36-81) for high-risk patients (OS p = 0.0075; TRM p = 0.0009).

**Conclusions:**

IR score at diagnosis of cGvHD predicts GvHD severity and overall survival. IR score may contribute to the risk stratification of patients. If confirmed in a larger and multicenter-based study, IR score could be adopted to identify patients at high risk and modulate cGvHD treatments accordingly in the context of clinical trial.

## Introduction

Allogeneic hematopoietic stem cell transplantation (HSCT) is a recognized curative treatment for several benign and malignant disorders. Although HSCT outcomes have improved significantly over time (1), long term survivors are at a defined relevant risk of developing complications; life expectancy remains lower compared to the age- and gender-matched population (2). Acute and chronic graft-versus-host disease (aGvHD and cGvHD, respectively) represent the most detrimental complications: with standard pharmacologic prophylaxis aGvHD occurring in 20-50% of patients and cGvHD in 30-50% (3). One third of cGvHD patients dies within 5 years of cGvHD diagnosis.

For more than three decades, high dose prednisone has been the only reliable therapy for cGvHD; however new drugs are now becoming available, and some have entered clinical practice with considerable success (4–6). Considering the recent availability of more treatment choices, the need for predictive and prognostic biomarkers has emerged.

In 2014, the National Institute of Health (7) defined criteria for developing GvHD biomarkers and their clinical role: I) prognostic biomarkers - to identify patients at high risk of cGvHD, II) diagnostic biomarkers - to help diagnosis in case of clinical uncertainty, and III) predictive biomarkers - to predict outcome and response to therapy.

Identifying reliable biomarkers in cGvHD is a difficult task due to the pleiomorphism of the disease, lack of sufficient patient numbers within prospective trials, but also technical issues such as difficulties in probes selection, availability of clinical grade tests, and time-points identification (8).

For its biological implications and for its feasibility, the assessment of immune reconstitution (IR) represents a good cGvHD biomarker candidate.

Previous studies have described associations between several cellular biomarkers and cGvHD (9–18), however no cGVHD cellular biomarker has yet been qualified for use in clinical applications (7).

In this study, we evaluated CD3+, CD4+, and CD8+ cells, NK cells, and B cells as well as immunoglobulins levels as potential predictive biomarkers of cGvHD, with the aim of defining an easy, reliable, and reproducible score to stratify patients at diagnosis of cGvHD.

## Materials and Methods

The primary endpoint of the study was to assess the impact of IR in risk stratification of cGvHD patients at diagnosis. The study objective was to find a prognostic index predicting the risk of TRM and probability of OS. To this aim we included additional cGvHD prognostic factors already identified by previous studies (19–21) in addition to IR variables.

### Patients

Patients aged >/= 18 years undergoing their first HSCT for any disease in indication and with any donor type or conditioning regimen, transplanted at IRCCS San Raffaele Scientific Institute between January 2011 and December 2016 were considered eligible for the study. Patients undergoing a second or third HSCT were excluded. A total of 411 patients met our inclusion criteria, among these 151 patients experienced cGvHD.

We first tested our score on a training set of consecutive patients undergoing HSCT between July 2012 and December 2016. Follow-up lasted until June 1, 2021 (or patients were censored earlier in case of a second HSCT). We then validated the scoring system retrospectively in all consecutive patients undergoing HSCT between January 2011 and June 2012 and who later developed cGvHD. Follow-up lasted until June 1, 2021. A second validation set to prospectively validate the IR score is under evaluation: patients transplanted between January 2017 and December 2019 are so far in follow-up, monitored for occurrence of cGvHD and classified according to IR score. The outcome analysis will be performed at the completion of the third year after HSCT of the last transplanted patients – December 2022 (**Supplementary Figure 1**).

### Prognostic Factors

We prospectively collected IR data of all our patients at the time of cGvHD diagnosis. IR variables were CD3+, CD3+CD4+, CD3+CD8+ (T cells and subsets), CD19+ (B cells), CD3-CD16+, and/or CD56+ (NK cells) absolute cell counts and levels of IgG, IgA, and IgM. The immunophenotype evaluation was performed on EDTA whole blood samples, using a lyse-no-wash technique and a panel of directly conjugated antibodies. Ten-color flow cytometry was performed using a Navios cytometer (Flow-Count™ Fluorospheres Beckman-Coulter) and Navios software. The single platform method was used to determine absolute counts. The analysis of lymphocyte subpopulations was performed on a lymphocyte population gate and on CD3+lymphocytes, using quadrant dot plot statistics. Immunoglobulin titers were assessed by immunoturbidimetric assays.

NIH 2004 (22) and subsequent 2014 (23) guidelines were followed for the diagnosis and staging of GvHD. Therapy and management followed our institutional protocol.

Clinical and transplantation variables (see below) used in the analysis included age, refined disease risk index (R-DRI) (24), HCT-Comorbidity Index (HCT-CI) (25), type of donor, GvHD prophylaxis, IR values at cGvHD diagnosis, history of prior acute GvHD, Karnofsky performance status (KPS), and platelet and total lymphocyte counts. These data and sample collection were part of the routine post-transplant assessment and did not require further blood sampling.

### Ethical Statement

In this non-interventional, prospective, observational cohort study, informed consent for the use of clinical data for scientific purposes was obtained from all patients undergoing HSCT in accordance with the Declaration of Helsinki.

All patients were treated according to current institutional programs upon written informed consent for transplant procedures, use of medical records, and immunological studies for patients undergoing allogenic HSCT within the non-interventional ALMON study, approved by San Raffaele Institutional Ethical Committee on October 19, 2007.

Data collection and storage were performed according to current institutional guidelines for ensuring privacy.

### Statistical Analysis and Definitions

The probability of overall survival (OS) was estimated using the Kaplan-Meyer estimator (26). Cumulative incidence was estimated for TRM to accommodate relapse as a competing risk. The log-rank test was used for univariate comparisons of survival curves, while the Gray’s test was conducted to compare cumulative incidences of competing risk endpoints. We built Cox multivariate models for OS in patients with cGvHD of any severity. Time was calculated from the development of cGvHD to the event of interest or last follow-up. Variables included in the models were the following: patient age (according to median value), R-DRI, type of donor (MRD – match related donor, MUD – match unrelated donor, CB – cord blood, MMRD – mismatch related donor), main GvHD prophylaxis (Anti Thymocyte Globulin [ATG]-based *vs* Post transplant Cyclophosphamide [PTCy]-based *vs* neither of the two), IR values at cGvHD diagnosis (according to median values), history of prior acute GvHD, Karnofsky performance status (KPS), platelet count <100x10^3^/mm^3^, total lymphocyte count <1.0 x 10^3^/mm^3^, and eosinophil count <0.5x10^3^/mm^3^. A backward stepwise procedure was used for variable selection with a p-value <0.05. Once we identified the variables independently predicting OS by multivariate analysis, we derived a formula for a prognostic risk index by using the β coefficients found in the model.

Each patient, for whom we had information about all the variables found in the model, was then assigned a numeric score and three groups of risk were identified (low, intermediate, and high) by dividing the population into three classes using the first and third quartiles. This choice was based on the assumption that the proportion of patients either at low or high risk would be lower than that of patients at intermediate risk. Finally, to evaluate predictive performance of the IR score, we calculated the receiver operating characteristics (ROC) curve and the area under the curve (AUC), to summarize the IR score ability to correctly classify events and non-events.

All statistical analyses were performed with the R software (R Development Core Team, Vienna, Austria).

## Results

### Patient Characteristics

Clinical features of patients with cGvHD are shown in **Table 1**. Among the 307 patients of the training set, 111 met the criteria for diagnosis of cGvHD according to NIH and among the 104 patients of the validation set, 40 met the criteria for diagnosis of cGvHD.

**Table 1:** cGVHD patients characteristics in the training and validation cohorts.

	Training cohort N = 111	Validation cohort N = 40	p
**Patient age, years, median [range]**	49 [17-77]	52 [19-72]	0.91
**Diagnosis, n (%)**			0.51
Acute leukemia	54 (49%)	22 (55%)	
MDS or MPN	19 (17%)	9 (22%)	
Lymphoma and myeloma	36 (32%)	8 (20%)	
Aplastic anemia	2 (2%)	1 (3%)	
**R-DRI at HSCT, n (%)**			0.44
Low-Intermediate	63 (56%)	25 (62%)	
High	43 (39%)	11 (28%)	
Very high	4 (4%)	3 (7%)	
Not applicable	1 (1%)	1 (3%)	
**HCT-CI score, median [range]**	2 [0-8]	0 [0-3]	<0.001
**Donor type, n (%)**			0.30
MRD	25 (22%)	14 (35%)	
MUD	34 (31%)	10 (25%)	
MMRD	52 (47%)	16 (40%)	
**Donor age, years, median [range]**	37 (18-73)	41 (19-58)	0.83
**Female donor/male recipient, n (%)**	31 (28%)	17 (42%)	0.11
**Host/donor CMV serostatus, n (%)**			0.86
pos/pos	76 (68%)	30 (75%)	
pos/neg	19 (17%)	6 (15%)	
neg/pos	3 (3%)	1 (3%)	
neg/neg	13 (12%)	3 (7%)	
**Conditioning intensity, n (%)**			0.008
RIC	26 (23%)	19 (48%)	
MAC	85 (77%)	21 (52%)	
**Stem cell source, n (%)**			0.34
BM	6 (5%)	0	
PB	105 (95%)	40 (100%)	
**GvHD prophylaxis**			<0.001
ATG-based	40 (36%)	29 (72%)	
PTCy-Sirolimus-based	56 (51%)	0	
Sirolimus-MMF	10 (9%)	5 (13%)	
CSA-MMF	2 (2%)	1 (3%)	
CSA-MTX	3 (3%)	5 (12%)	

MDS, myelodysplasia; MPN, myeloproliferative neoplasms; R-DRI, revised disease risk index; HCT-CI score, Hematopoietic Cell Transplantation – specific Comorbidity Index; MRD, match related donor; MUD, match unrelated donor; MMRD, mismatch related donor; CMV, cytomegalovirus; RIC, reduced intensity conditioning; MAC, myeloablative conditioning; BM, bone marrow stem cells; PB, peripheral blood stem cells; MMF, micophenolate mofetil; CSA, cyclosporine-A; MTX, methotrexate; ATG, antithymocyte globulin; PTCy, post-transplant cyclophosphamide.

The two cohorts were similar for age, sex, disease type, graft source, R-DRI at transplant, level of mismatch, and CMV serostatus. Compared to the training cohort, the validation set included a lower proportion of patients receiving myeloablative conditioning (MAC) (52% *vs* 77% - p 0.008), a higher proportion of patients receiving ATG as GvHD prophylaxis (ATG 72% *vs* 36%) with no patients receiving PTCy, against 51% of patients in the training cohort (p <0.001). Finally, the HCT-CI score was lower in the validation cohort than in the training one (p <0.001).

Almost half of the patients received a transplant from a haploidentical family donor (47% in the training set, 40% in the validation cohort, ns).

GvHD prophylaxis in the training cohort relied mainly upon ATG in the MUD setting and on PTCy + sirolimus in haploidentical transplants, while in the validation cohort ATG was the backbone of GvHD prophylaxis both for MUD and MMRD. Peripheral blood was the preferred stem cell source in both cohorts. The proportion of MRD/MUD/MMRD was equally distributed across patients with or without cGvHD in both sets.

Median follow-up was 6 years [range 1 - 8.5] in the training set and 9.2 years [6.4 – 10] in the validation set. Median time to GvHD was 198 days [range 32-926] in the training set and 161 days [range 39-1304] in the validation set.

In the training set, the 2-year OS and 2-year cumulative incidence of TRM from cGvHD diagnosis were 71% (95% CI, 61-79) and 13% (95% CI, 7-20), respectively. In the validation set, the 2-year OS and 2-year cumulative incidence of TRM were 73% (95% CI, 56-84) and 23% (95% CI, 11-37), respectively.

### Chronic GvHD Features According to NIH Classification

In the training set, the 3-year cGvHD incidence was 35% (95% CI, 29-40%) with 27% moderate-severe cGvHD (95% CI, 22-32%), while in the validation set, it was 36% (95% CI, 27-45) with 33% moderate-severe (95% CI, 24-42).

According to NIH definition, there were 69 (62.2%) classic-type cGvHD (21 mild, 26 moderate, 22 severe), and 42 (37.8%) overlap cGvHD (5 mild, 15 moderate, 22 severe) in the training cohort and 21 (52.5%) classic-type cGvHD (2 mild, 9 moderate, 10 severe), and 19 (47.5%) overlap cGvHD (0 mild, 5 moderate, 14 severe) in the validation cohort. Of note, 37 patients (33%) in the training cohort and 21 (52%) in the validation cohort were previously diagnosed with acute GvHD.

All patients with a diagnosis of cGvHD were treated at our long-term follow-up clinic according to institutional guidelines and EBMT recommendations (27). All patients with a moderate to severe cGvHD received first line treatment with high-dose prednisone (0, 5-1 mg/Kg), topical therapy was added when appropriate.

### Immune Reconstitution as Predictive Factor for cGvHD—Algorithm Development and Validation

The following variables independently predicting OS at cGvHD diagnosis were identified: CD4+ count >233 cells/mm^3^ (β 3.09, p 0.01), NK count <115 cells/mm^3^ (β 1.75, p 0.02), IgA <0.43 g/L (β 1.47, p 0.03), IgM <0.45 g/L (β 2.22, p 0.007), Karnosky PS <80% (β 5.05, p <0.001), and PLT <100x10^3^/mm^3^ (β 2.18, p 0.02). The multivariate Cox regression analysis of factors determining OS is reported in **Table 2**.

**Table 2:** Multivariate Cox-regression analysis of factors determining OS.

	OS		
	HR (95% CI)	β coefficient	p
**CD3+CD4+ cells/mm^3^ at cGvHD diagnosis**			
≥233 cells/mm^3^ Vs <233 cells/mm^3^	21.9 (1.9-57)	3.09	0.014
**NK cells/mm^3^at cGvHD diagnosis**			
<115 cells/mm^3^ Vs ≥115 cells/mm^3^	5.7 (1.4-23)	1.75	0.017
**IgM at cGvHD diagnosis**			
<0.45 g/L Vs ≥0.45 g/L	9.2 (1.8-36)	2.22	0.007
**IgA at cGvHD diagnosis**			
<0.43 g/L Vs ≥0.43 g/L	4.4 (1.13-16.7)	1.47	0.032
**Karnofsky PS at cGvHD diagnosis**			
<80% Vs ≥80%	72 (12-421)	5.05	<0.001
**Platelet counts at cGvHD diagnosis**			
<100 x10^3^/mm^3^ Vs ≥100x10^3^/mm^3^	8.83 (1.3-58)	2.18	0.024

Covariates included in the model: Patient age (according to median value), R-DRI, type of donor (MRD, match related donor; MUD, match unrelated donor; CB, cord blood; MMRD, mismatch related donor), main GvHD prophylaxis (Anti Thymocyte Globulin [ATG]-based vs Post transplant Cyclophosphamide [PTCy]-based vs neither of the two), IR values at cGvHD diagnosis (according to median values), history of prior acute GvHD, Karnofsky performance status (KPS), platelet count <100x10^3^/mm^3^, total lymphocyte count <1.0 x 10^3^/mm^3^, and eosinophil count <0.5x10^3^/mm^3^.

IR parameters at time of cGvHD onset are reported in **Table 3**. In the training cohort, the median time of IR parameters evaluation was 189 days. Overall, the median time of collection of IR parameters was 150 days.

**Table 3:** Immune reconstitution parameters at diagnosis of cGvHD.

Parameter	Median value [range]
**CD3+**	706 cells/mm^3^ [53-6132]
**CD3+CD4+**	233 cells/mm^3^ [15-1642]
**CD3+CD8+**	470 cells/mm^3^ [13-5094]
**CD19+**	21 cells/mm^3^ [0-1206]
**IgG**	4.22 g/L [0-29.83]
**IgA**	0.43 g/L [0-5.42]
**IgM**	0.45 g/L [0-5.11]
**NK**	115 cells/mm^3^ [16-991]

An algorithm was created based only on variables that predicted OS significantly and independently, i.e., CD4+ count >233 cells/mm^3^, NK count <115 cells/mm^3^, IgM <0.45 g/L, IgA <0.43 g/L, Karnosky PS <80%, and PLT <100x10^3^/mm^3^. To calculate the final score, we took into account the different weight of these six variables in predicting OS, expressed by their beta coefficient. The final score was calculated as follows:

3.09 (if CD4 > 233 cells/mm^3^ at time of cGvHD diagnosis) + 1.75 (if NK < 115 cells/mm^3^ at time of cGvHD diagnosis) + 1.47 (if IgA < 0.43 g/L at time of cGvHD diagnosis) + 2.22 (if IgM < 0.45 g/L at time of cGvHD diagnosis) + 5.05 (if Karnofsky <80 at time of cGvHD diagnosis) + 2.18 (if PLT <100x10^3^/mm^3^ at time of cGvHD diagnosis).

Each function in the parenthesis is considered 1 if the condition is satisfied, or otherwise 0.

We then calculated the IR score for 87 patients of the training set (24 were excluded because of missing data). The 25^th^ quartile value was 3.09, the 75^th^ one was 6.91: low-risk patients were defined as having a score ≤3.09, intermediate as having a score >3.09 and ≤6.91, and high risk as having a score >6.91.

Patients’ distribution according to NIH consensus classification and according to IR score is presented in **Table 4**. Additional information is provided in **Supplementary Figure 2**.

**Table 4:** Cross-stratification of cGvHD patients into respective risk groups by NIH consensus and IR score.

	Training cohort			Validation cohort			Total
	*IR low risk*	*IR int risk*	*IR high risk*	*IR low risk*	*IR int risk*	*IR high risk*	
*NIH consensus mild*	12	10	3	0	1	1	27
*NIH consensus moderate*	7	17	6	5	3	6	44
*NIH consensus severe*	5	14	13	5	4	15	56
Total	24	41	22	10	8	22	127

IR, immune reconstitution score; int, intermediate.

**Figure 1:**
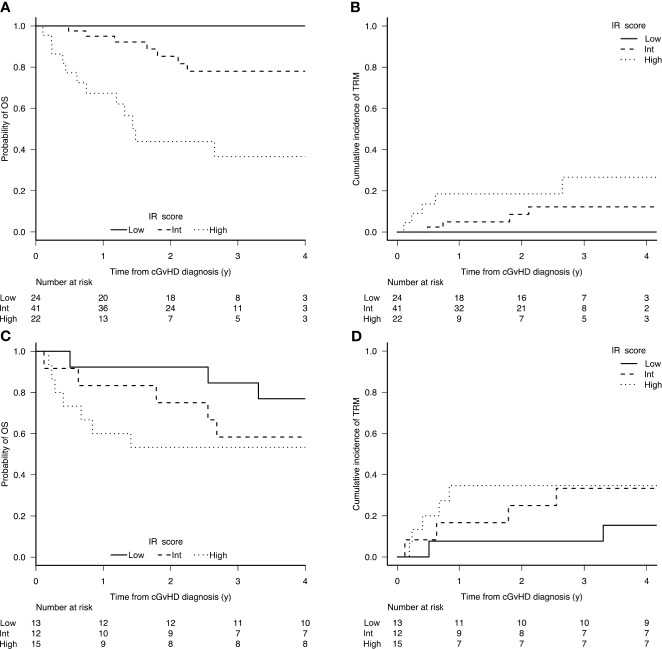
Cumulative incidence of OS **(A)** and TRM **(B)** according to the prognostic score in the training set (n 87); and OS **(C)** and TRM **(D)** in the validation set (n 40).

In the training set, the 6-year OS and TRM were stratified by both IR score and NIH consensus classification. The 6-year OS and TRM by IR score were 85% (95% CI, 70-92) and 13% (95% CI, 5-25) for low-risk patients, 64% (95% CI, 44-89) and 30% (95% CI, 15-47) for intermediate-risk patients, and 26% (95% CI, 10-47) and 42% (95% CI, 19-63) for high-risk patients (OS p<0.0001; TRM p = 0.015, **Figures 1A, B**). The 6-year OS and TRM by NIH consensus classification were 87% (95% CI, 65-96) and 9% (95% CI, 1-25) for mild cGvHD, 68% (95% CI, 51-80) and 20% (95% CI, 9-33) for moderate cGvHD, and 49% (95% CI, 33-64) and 44% (95% CI, 28-59) for severe cGvHD (OS p = 0.009; TRM p = 0.005).

In the validation set, the stratification according to IR score was confirmed to be significant, while the stratification according to NIH consensus was clearly significant for TRM and showed a trend for OS. The 6-year OS and TRM by IR score were 83% (95% CI, 48-96) and 8% (95% CI, 1-32) for low-risk patients, 78% (95% CI, 37-94) and 11% (95% CI, 1-41) for intermediate-risk patients, and 37% (95% CI, 17-58) and 63% (95% CI, 36-81) for high-risk patients (OS p = 0.0075; TRM p = 0.0009, **Figures 1C, D**). The 6-year OS and TRM by NIH consensus classification were 100% and 0% for mild cGvHD, 71% (95% CI, 41-88) and 14% (95% CI, 2-38) for moderate cGvHD, and 48% (95% CI, 27-67) and 51% (95% CI, 29-70) for severe cGvHD (OS p = 0.157; TRM p = 0.0332).

**Figure 2:**
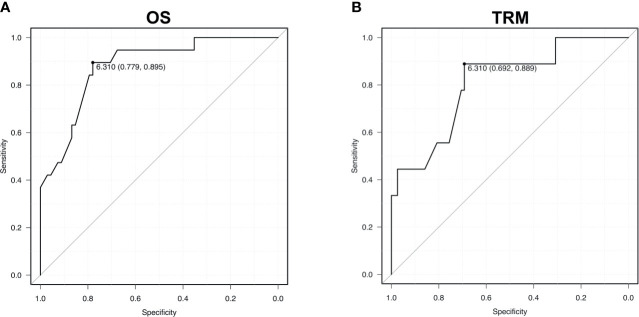
Receiver operating characteristic (ROC) curves for OS **(A)** - area under the curve 0.88 - and TRM **(B)** – area under the curve 0.81. AUC values are reported from multivariable models.

To support the validity of the IR score, the ROC curve *via* the AUC was calculated: AUC values were 81% for TRM and 88% for OS. A cut-off of 6.310 was identified with 69% sensitivity and 89% specificity for TRM, and 78% sensitivity and 90% specificity for overall mortality (**Figure 2**).

### IR Score Stratifies Patients Independently From NIH Consensus cGVHD Criteria

The low-risk group included 24 and 10 patients in the training set and validation set, respectively, while the intermediate-risk group included 41 and 8 patients, and the high-risk group 22 and 22 patients.

We challenged the capability of our IR score of stratifying patients across the different NIH clinical stages (**Table 4**).

In the training cohort, the 2-year OS from cGvHD diagnosis for patients with mild cGvHD (n = 25) according to NIH classification was 100% for low and intermediate and 33% (95% CI, 1%-77%) for high-risk IR score (p <0.001). The 2-year OS for moderate cGvHD patients (n = 30) stratified according to the IR score was 100%, 83% (95% CI, 46-96%), and 62% (95% CI, 14%-89%) in low, intermediate, and high-risk groups, respectively (p 0.16). For severe cGvHD patients (n = 32), 2-year OS was 100%, 76% (95% CI, 41-92%), and 40% (95% CI, 13%-66%) in low, intermediate, and high-risk groups, respectively (p 0.02). Results therefore confirmed the independent stratification within cGVHD clinical grades.

### IR Score Predicts cGVHD Mortality

We next evaluated the contribution of the IR cGVHD score in predicting TRM. Chronic GvHD was the cause of death in 2, 1, and 12 patients classified as low, intermediate, and high-risk according to IR score. High-risk patients were more likely to die from cGVHD than low and intermediate-risk patients (p<0,0001). No patients died due to infection in the low-risk group, while 9 and 3 patients died due to infectious complications in the intermediate and high-risk groups, respectively (p ns).

## Discussion

Chronic GvHD represents one of the major hurdles in the management of HSCT survivors. Despite progress in the optimization of conditioning regimens, ancillary measures, and pre-emptive strategies for infectious complications, we are still facing the unmet medical need of cGvHD treatment. cGVHD is responsible for 30% to 50% of non-relapse mortality in long-term survivors (28). According to data from the Fred Hutchinson Cancer Research Center (29), only approximately 50% of cGvHD patients are cured within 7 years after starting systemic treatment, 10% require continuous treatment, and 40% die within 7 years. Moreover, at 5 years from cGvHD diagnosis, only 32% of patients are alive, free of immunosuppressive therapy, and in complete remission from the primary disease (30).

The identification of valid and reproducible biomarkers for both acute and chronic GvHD is one of the most significant challenges in the field. While clinical trials investigating new drugs for the treatment of acute GvHD nowadays are designed according to patients’ stratification based on established biomarkers, this is not the case for cGvHD. cGvHD is characterized by pleiomorphic manifestation and a complex pathogenesis that elicits both inflammatory and fibrotic pathways. cGvHD affects more than one third of transplanted patients and clinical presentation at onset only partially unveils the true severity of the disease. Clinical grading, including the latest NIH consensus criteria, is not able to provide univocal prognosis of such a complication.

The identification of patients at risk is mandatory for correct cGvHD management. While innovative, highly effective, but also toxic drugs are released on the market, early identification of high-risk patients—at the time of cGvHD diagnosis—would enable an earlier and more aggressive therapy while sparing toxicity to low-risk patients. So far, biomarker studies are in progress to identify tools to enhance diagnosis and definition of prognosis, however results are still far from routine practice.

While acute GVHD is mediated by mature effector T cells from the donor (graft) that become activated after encountering alloantigens in the recipient, cGVHD is characterized by aberrant immune responses to both autoantigens and alloantigens (31, 32). Chronic GvHD arises from a failure to develop tolerance after HSCT (33). The loss of regulator-cell function appeared to be one of the critical events in the development of cGVHD: aberrant B – T – NK cells homeostasis and the inability to establish cell tolerance is a pivotal point of cGvHD (33–35). A recent international multicenter study in children and adolescents provided new insights on the immune profile peculiarity of cGvHD (33). In cGvHD, decreased transitional B cells and increased cytolytic NK cells are associated with increased activated T cells, naive helper T, and cytotoxic T cells, loss of regulatory NK cells, and increased ST2 and soluble CD13. The immune signature of cGVHD is complex with several cytokine, T-cell, NK-cell, and B-cell abnormalities (33–35). Definition of immune-based biomarker algorithms will assist in assigning patient risk for cGVHD, with the possibility of a risk-tailored treatment approach (33).

We investigated IR as a candidate biomarker, using easily collectable variables, with a high grade of reproducibility and standardization within a setting of well-known clinical grade tests. The overall incidence of cGvHD in our patient population was similar to that reported in the literature, moreover all the available HSCT platforms in terms of donor selection (MRD, CB, MUD, MMRD) and GvHD prophylaxis (ATG-based, cyclosporin-based, rapamycin-based, and PTCy-based) were represented adequately, providing an additional strength to the study.

The IR score-based algorithm provided a risk stratification power that proved independent from the nature of both GvHD prophylaxis and donor source in both the training set and in the validation cohort.

We had the opportunity to analyze over 100 consecutive cGvHD patients with an adequate follow-up. Strengths of our study were the prospective sample and data collection, the homogeneous management of post-HSCT follow-up, and the systematic clinical evaluation of patients for GvHD according to NIH guidelines. Being a single-center study, cohort size was limited and suggests the need of further validation in multicenter cohorts.

Our results showed a clear impact of immunological variables at cGvHD diagnosis: CD3+CD4+ counts, NK cells, and IgA and IgM levels were selected by our model over other clinical variables as independent predictors of patient outcome. Very few studies have demonstrated an association between biological markers and survival; more information has been found regarding biomarkers for the prediction of cGvHD risk and has been associated with the diagnosis of cGvHD (7, 36).

In addition, the IR approach has highlighted some interesting biological pathways:

- In the risk score we generated, higher CD3+CD4+ (>233 cells/mm^3^) counts are linked to worse outcome. This may seem counterintuitive as the main cause of death in cGvHD patients is infection due to immunosuppression. But considering we are analyzing the cell count at the onset of cGvHD, this may reflect the pathophysiologic role of CD4+ T helper cells in cGvHD pathogenesis. In their recent review of cGvHD pathophysiology (3), Zeiser and Blazar describe the role of CD4+ cells in orchestrating the dysregulated immune response after an initial injury. Ibrutinib, the only FDA-approved drug for steroid-resistant cGvHD, targeting Bruton’s Tyrosin Kinase (in the path of B cell activation) and inducible-T cell kinase (in the path of T helper cell activation), showed good response rates [67%, in a phase II multicenter study by Miklos and colleagues (5)]. T cell depletion (linked to slower kinetics of IR) is associated with lower rate of chronic GvHD (36, 37). Evidence suggests that high CD4+ counts at GvHD diagnosis may indeed reflect a strong initial orchestrating signal for cGvHD. CD4+ counts have been investigated as prognostic biomarkers by several studies with somewhat contradictory results. However, these studies did not test CD4+ counts at onset of cGvHD. Independently from cGvHD, in transplanted patients, a fast and robust recovery of CD4+ counts at early time-points after HSCT was associated with low TRM (38, 39). This is possibly linked to the protection from opportunistic infections mediated by T cells early after transplant. High CD4+ counts have already been associated with acute GvHD (40, 41). Importantly, Podgorny and coworkers observed a persistently higher number of CD4+ counts after HSCT in patients developing cGvHD requiring systemic therapy than in cGvHD patients who did not require systemic treatment, in line with our results.
- NK cells were found to have a negative prognostic implication when lower than 115 cells/mm^3^. This finding points to the protective effect that NK cells have in cGvHD pathophysiology; it was demonstrated (42) that NK cells mediate the reduction of GvHD by inhibiting activated, alloreactive T cells while retaining graft-versus-tumor effects through effector molecules such as FasL (43). Thus, similarly to T cells, NK cells display a potent anti-leukemia effector capacity, and yet, unlike them, do not mediate cGvHD (44). In the context of haploidentical transplantation performed within a PTCy regimen (45), the percentage of alloreactive mature NK cells quantified after transplant negatively correlated to relapse risk but not to cGvHD rate. Noticeably, NK cells are critical players of innate immunity against viral and bacterial infections at the mucosal barriers (46). We can thus speculate that cGvHD patients with high NK cell levels may benefit from this effect, resulting in improved outcome. In the above-mentioned study, Podgorny et al. (40) showed reduced levels of regulatory NK cells in patients with severe cGvHD compared to those not requiring systemic therapy. In several studies, high NK cell counts early after HSCT have been associated with low TRM and low aGvHD incidence, in both HLA-matched and HLA-mismatched transplant settings (47–49).
- Low IgM and IgA levels were the last IR variables significantly associated with worse prognosis in our cGvHD patient cohort. B cells reconstitution occurs relatively late after HSCT. Post-transplant B cell deficiency is—at least in part—due to insufficient B lymphopoiesis and in part, this is exerted by GvHD (50). The pathogenic role of B cells in cGvHD was first identified in murine models in 1995 (51). Recently, dysregulated B cell lymphopoiesis was proven to be associated with the onset of chronic GvHD (52). Immunoglobulin levels seem to recover in parallel to B cell reconstitution, in which recovery of Ig subclasses usually occurs in a distinctive order (53). After HSCT, Ig levels drop reflecting the absence of Ig-producing B cells. As a reflection of normal ontogeny, IgM production will reconstitute relatively early, subsequently IgG generally reaches normal levels, whereas normalization of IgA levels may take longer. Chronic GvHD is associated with significantly poorer B cell reconstitution in both function and numbers. IgM levels were consistently low in cGvHD patients and our result was in line with previous pubblications (10, 35). Khoder et al. (54) demonstrated that regulatory B cells (enriched in IgM subsets) are deficient in cGvHD patients. Abdel-Azim et al. (55) reported that IgM memory B cells were persistently lower within the first two years after HSCT in cGvHD patients, than in transplant recipients not developing cGvHD.

All these findings support the items in our prognostic score impacting cGvHD outcome. The validation step performed on the retrospective cohort is also encouraging. The score held its power in an independent cohort, despite the differences in conditioning and prophylaxis strategies. This suggests a link of the proposed score with cGvHD pathogenesis and progression, events triggered with different frequencies by different transplant platforms, but possibly similar once the disease is established.

The current study adds a new insight to a big research area on prognostication of cGvHD, going beyond scoring systems only based on clinical parameters. Clinical classification according to NIH consensus criteria displays a clear stratification for both OS and TRM; IR score was able to provide an additional stratification to implement the prognostic power at cGvHD declaration. IR score highlights among each clinical class the long-term probability of survival.

We can confirm that both IR-score stratification and NIH categorization were able to independently prognosticate TRM and OS. NIH categorization keeps its relevance but is not 100% accurate in identifying all high or low-risk patients; the IR-score biomarkers help in selection of high and low-risk patients also within their NIH risk groups. Still, in the majority of cases, there was concordance between clinical risk and IR risk, thus our results are not in contrast with the known prognostic impact of NIH categorization of cGVHD. Overall, patients with severe GvHD according to NIH classification have worse OS and TRM compared to mild GvHD, but among patients with severe GvHD those with a low-risk IR score have better prognosis in terms of OS and TRM. Similarly, patients with mild/moderate GvHD present better OS and TRM overall, but the IR score was able to predict patients at high risk of progression towards severe forms and—ultimately—worse outcome.

This suggests that the IR score can improve prognostication, especially if combined with clinical staging. Beyond the use as a definite prognostic tool, our IR score proved the important role of IR in the clinical management of cGvHD patients, suggesting further research as well as systematic clinical application of IR monitoring programs and IR-based therapeutic decisions.

Of note, we recognize that in the training cohort a consistent proportion of patients received, as GvHD prophylaxis, a combination of pTCy and rapamycin. This combination is peculiar and is not a standard one, but also other platforms were well represented in the patient population. The current results should be confirmed in a multicenter study as well as with longer follow-up and expansion of the sample size.

We conclude that an IR-based algorithm represents a valid tool to identify high-risk patients at cGvHD onset. The algorithm predicts long-term OS and TRM, identifying subjects at high risk of death due to cGvHD through stratification into three classes of risk and the clear identification of a cut-off strongly associated with both overall mortality and TRM.

Future directions should include prospective and serial evaluations of the algorithm to define its clinical use. Our goal for the next years will be to identify tools able to shape the treatment options not only according to clinical presentation but also to risk stratification at the onset of such a detrimental transplant complication.

## Data Availability Statement

The raw data supporting the conclusions of this article will be made available by the authors, without undue reservation.

## Ethics Statement

The studies involving human participants were reviewed and approved by San Raffaele Institutional Ethical Committee. The patients/participants provided their written informed consent to participate in this study.

## Author Contributions

Conception, design, and manuscript revision: ML-S, FS, FLo, and FC. Provision of study material or patients: all authors. Collection and assembly of data: ML-S, FS, and FLo. Data analysis and interpretation: ML-S, FS, FLu, and FC. Manuscript writing: ML-S, FS, FLo, SMar, and FC. Final approval of the manuscript: all authors. All authors contributed to the article and approved the submitted version.

## Conflict of Interest

The authors declare that the research was conducted in the absence of any commercial or financial relationships that could be construed as a potential conflict of interest.
