# Supplementary material for: Immune Reconstitution-Based Score for Risk Stratification of Chronic Graft-Versus-Host Disease Patients
Source: Front Oncol. 2021 Jul 22;11:705568. doi: 10.3389/fonc.2021.705568 (PMC8341942; doi:10.3389/fonc.2021.705568)
Supplement: Supplementary file 1 [file DataSheet_1.pdf]

Supplementary Material

Supplementary Figure 1 - Scheme of algorithm development and validation

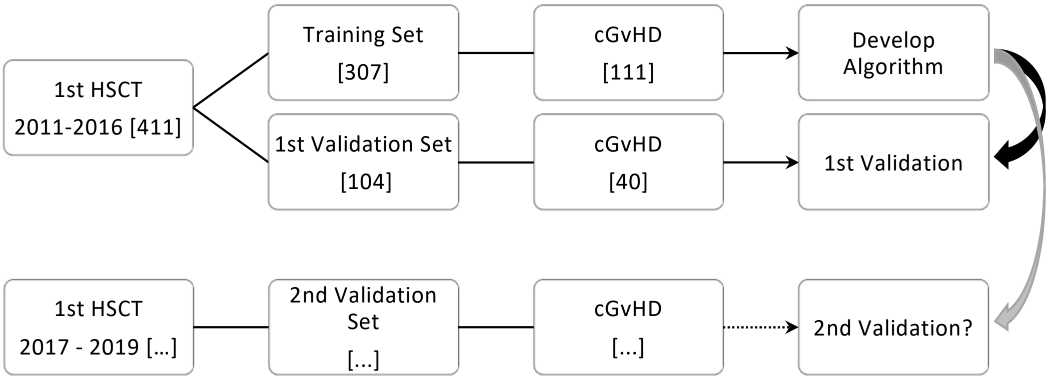

Supplementary Figure 2 – Stratification of patients (training cohort + validation cohort) according to NIH consensus and IR score risk. Panel A patients classified with mild NIH cGvHD. Panel B patients classified with moderate NIH cGvHD. Panel C patients classified with severe NIH cGvHD.

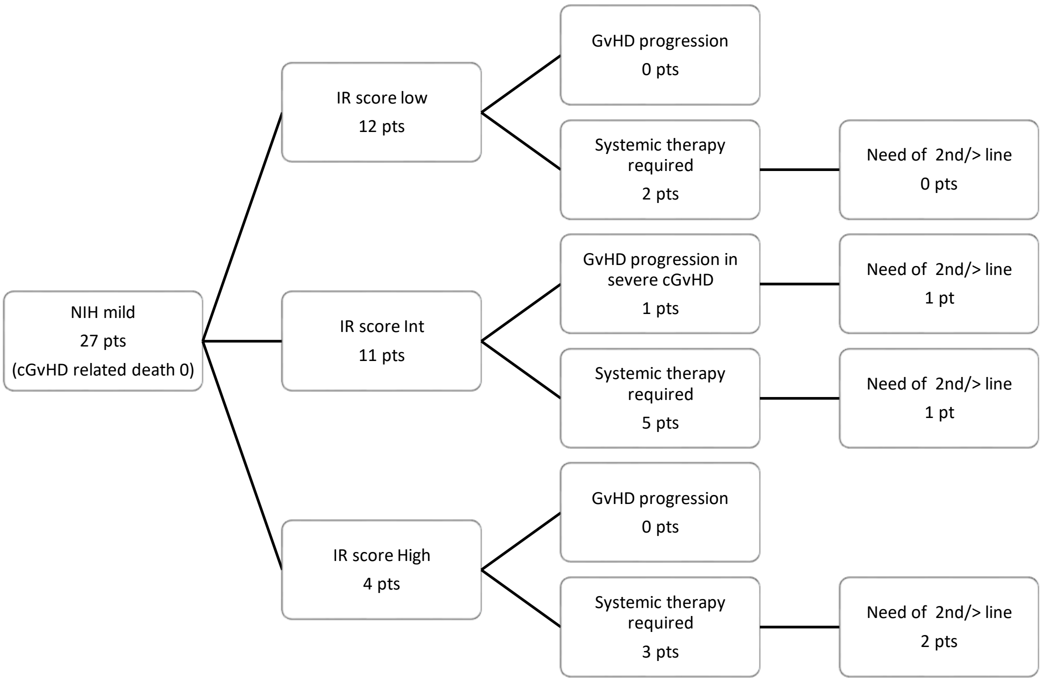

A

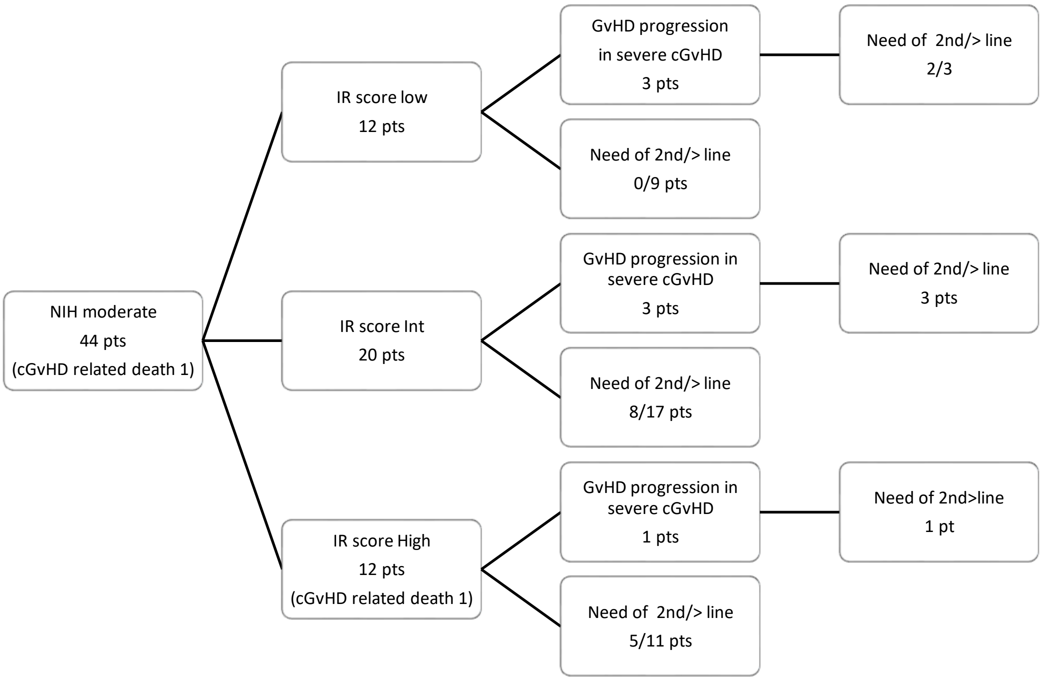

B

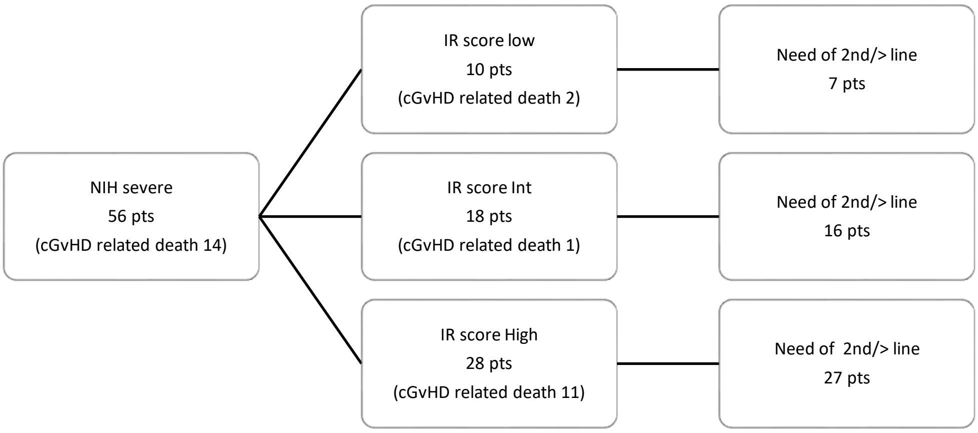
NIH severe
56 pts
(cGvHD related death 14)
IR score low
10 pts
(cGvHD related death 2)
IR score Int
18 pts
(cGvHD related death 1)
IR score High
28 pts
(cGvHD related death 11)
Need of 2nd/> line
7 pts
Need of 2nd/> line
16 pts
Need of 2nd/> line
27 pts

C
